# Adjusting plant nutrient acquisition to fluctuating availability: transcriptional co-regulation of the nitrate and phosphate deprivation responses in roots

**DOI:** 10.1093/jxb/erab131

**Published:** 2021-05-04

**Authors:** Uwe Ludewig, Emil Vatov, Dominik Hedderich, Benjamin Neuhäuser

**Affiliations:** Institute of Crop Science, Nutritional Crop Physiology, University of Hohenheim, Fruwirthstr., Stuttgart, Germany

**Keywords:** Nitrate deprivation, nitrogen starvation, phosphate deprivation, reactive oxygen species

## Abstract

This article comments on:

**Safi A, Medici A, Szponarski W, Martin F, Clement-Vidal A, Marshall-Colon A, Ruffel S, Gaymard F, Rouached H, Leclercq J, Coruzzi G, Lacombe B, Krouk G**. 2021. GARP transcription factors repress Arabidopsis nitrogen starvation response via ROS-dependent and -independent pathways. Journal of Experimental Botany **72**, 3881–3901.

**A family of NIGT1/HHO-type transcriptional repressors activate phosphate deprivation responses via inhibition of upstream repressors. At the same time, these repress the nitrate deprivation response upon nitrate provision; their loss triggers an increase of nitrate uptake and plant growth. Safi *et al.* (2021) link the function of NIGT1/HHOs with reactive oxygen species (ROS) signalling under variable nitrate supply, but it remains puzzling how ROS integrate into nutrient-specific signalling cascades.**

Native plant ecosystems are often nutritionally co-limited, especially in the two macroelements nitrogen (N) and phosphorus (P) (Fay *et al.*, 2015). Substantial biomass increases are only expected when both elements are added, indicating synergisms between them. On evolutionary scales, nutritional deficiencies were the rule, rather than the exception, and modern heavily fertilized crops carry relicts of this in their genetics. Different plant species have different traits related to the acquisition of inorganic phosphate (P_i_, one of the least soil-mobile nutrients that is easily sorbed and fixed to clay particles) and nitrate (a highly mobile nutrient in soil), but common responses to low P and N exist, such as the anthocyanin production in leaves and the investment into roots, which results in a higher root/shoot ratio, mediated by phytohormone-driven redirection of photoassimilates. Nutritional interactions occur with other elements as well; for example, molybdenum and iron are linked with nitrate reduction, whereas P_I_ mobilization mechanisms often co-solubilize iron, zinc, and manganese (Marschner, 2011).

## Function of NIGT1/HHOs in the P_i_ deprivation response and the link with nitrate

**Fig. 1.**
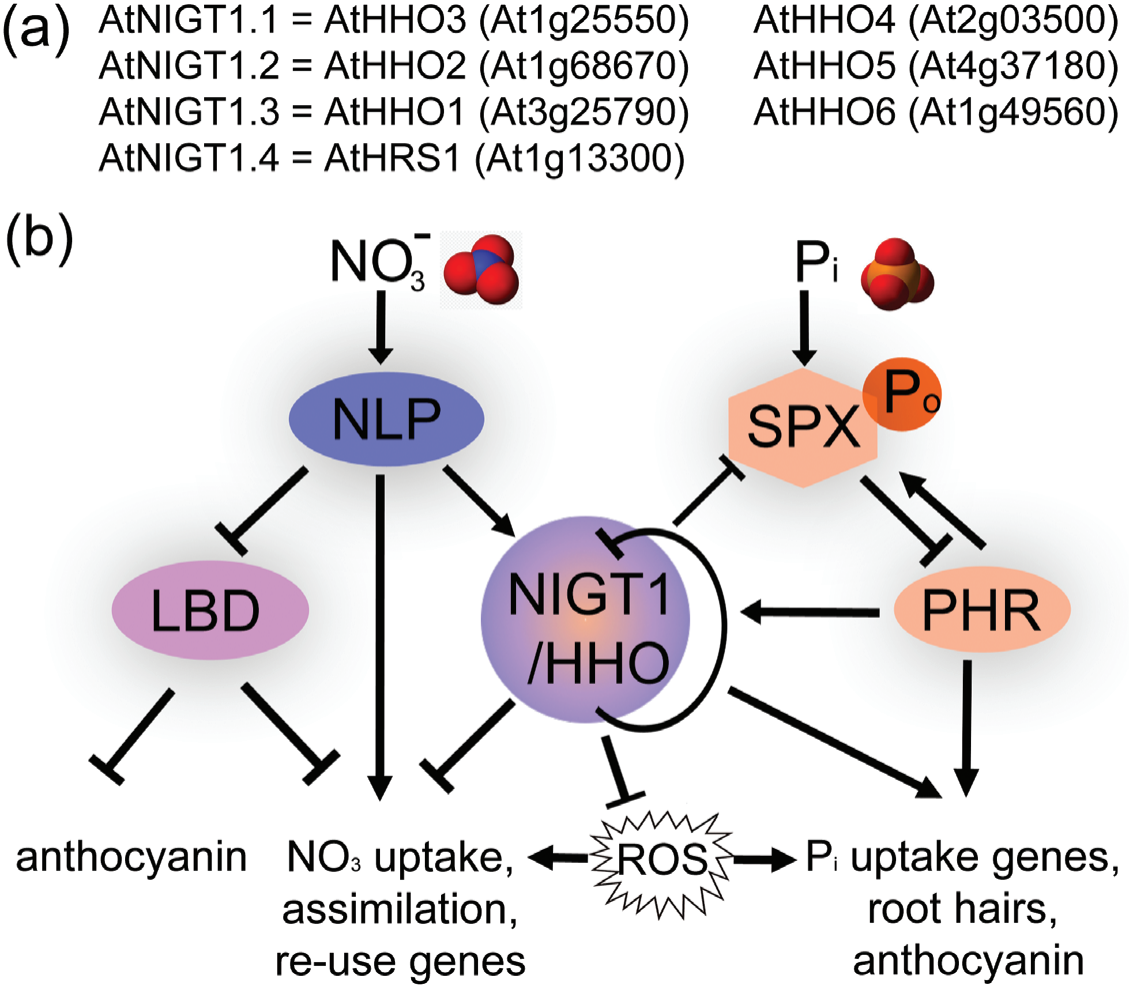
*NIGT1/HHO* gene IDs, NO_3_^–^ and P_i_ signalling cascades, and the link with ROS. (A) Nomenclature of *NIGT1/HHO* repressors. Note that all four *NIGT1* transcripts are up-regulated by nitrate, with *NIGT1.3* and *NIGT1.4* most nitrate responsive. (B) Simplified scheme of NIGT1/HHO regulation in nutrient deprivation responses. The diagram shows the signalling cascades upon the presence of nitrate and/or phosphate. For gene abbreviations see text.

*NIGT (NITRATE-INDUCIBLE GARP-TYPE TRANSCRIPTIONAL REPRESSOR)* genes, also known as *HRS1 (HYPERSENSITIVITY TO LOW P*_*i*_*-ELICITED PRIMARY ROOT SHORTENING 1*), and *HHO* (*HRS1 HOMOLOG*) genes coordinate nitrate and P_i_ responses in Arabidopsis (Kiba *et al.*, 2018; Maeda *et al.*, 2018; Ueda et al., 2020b, c; Wang *et al.*, 2020). Their names already indicate that they were identified in different nutritional contexts (Fig. 1).

*NIGT1/HHO* genes act as positive regulators of the P_i_ starvation response. In brief, the master upstream regulators PHOSPHATE STARVATION RESPONSE 1–4 (PHR1–PHR4) bind to *cis*-elements in their promoters and activate them. In the presence of P_i_, PHRs form inactive complexes with SPX proteins (named after SYG1/Pho81/XPR1); the latter apparently sense the cellular P status. Under P_i_ deficiency, PHRs are released and target *cis*-elements in promoters of P_i_ uptake-, recycling-, and morphological adaptation-associated genes (Fig. 1). NIGT1/HHOs enhance transcription of P_i_ uptake transporters (Wang *et al.*, 2020) and repress SPX promoters, while PHRs moderately activate *NIGT1/HHO* genes (Maeda *et al.*, 2018).

NIN-LIKE PROTEIN 6/7 (NLP6/7) transcription factors, master regulators of the nitrate response, promote nitrate uptake/assimilation genes and *NIGT1* expression (Fig. 1). NIGT1/HHOs dimerize with each other and with more distant HHO proteins; this determines specificity and affinity for targets (Ueda *et al.*, 2020b).

**Fig. 2.**
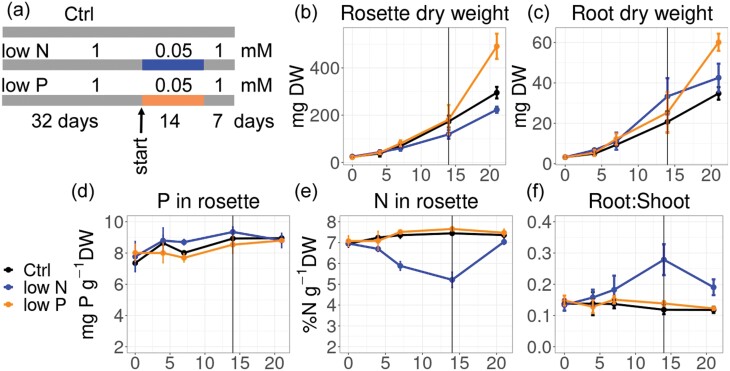
Biomass boost by fluctuating nutrient supply: release of growth inhibition after transient decrease of P_i_ supply in hydroponics. (A) Arabidopsis Col-0 plants were grown in standard nutrient solutions (Menz *et al.*, 2018) with 1 mM ammonium nitrate and P_i_ for 52 d (control), or 32 d and then transiently transferred to 50 µM N (low N) or 50 µM P_i_ (low P) for 2 weeks. These plants were then resupplied with full nutrient solution with 1 mM P_i_ and N for an additional week (all other nutrients were kept at the same level). (B) Shoot biomass, (C) root biomass, (D) P in rosette, (E) N in rosette, (F) root/shoot ratio. Note that the growth was transiently impaired in low N, but not in low P (as P_i_ was still sufficient for maximal growth). After the transient treatment, ‘low P’ plants grew much better than controls that achieved high P throughout. This is an example showing that nutrient fluctuations, if not growth limiting, can have beneficial effects on plant growth (and possibly nitrate acquisition). It is possible, but not known, whether NIGT1/HRS signalling is responsible for this effect. We note that experiments under laboratory conditions with agar plate/hydroponic systems often involve P_i_ concentrations >100-fold higher than typical soil P_i_ solution concentrations (<10 µM P_i_).

In a feedback loop, NIGT1/HHO proteins repress nitrate uptake upon P_i_ deprivation, and vice versa. P_i_ uptake is repressed in the absence of nitrate, as *SPX* promoters are then not repressed by NIGT1/HHOs (Ueda *et al.*, 2020c). Safi *et al.* showed that double and quadruple *nigt1/hho* mutants had elevated high affinity nitrate uptake concomitant with the de-repression of high affinity nitrate transporters. This correlates with an improved growth under controlled conditions with sufficient P_i_ (Safi *et al.*, 2021). Wang *et al.* (2020) also previously reported an improvement of low affinity uptake in *hho* mutants, probably due to NRT1.1. One should, however, keep in mind that Col-0, the genotype used in these studies, is quite inefficient using nitrogen compared with other Arabidopsis accessions (Chardon *et al.*, 2010; Menz *et al.*, 2018). Even though there is functional diversification within the NIGT1/HHO family among monocots and dicots (Ueda *et al.*, 2020a), the transcriptional co-repression of nitrate influx by P_i_ deficiency seems to be conserved in maize (Wang *et al.*, 2020), and P_i_ starvation control by nitrate is conserved in wheat and rice (Medici *et al.*, 2019). Still, extrapolation to field-grown crops should be done with caution, as biomass increases under control conditions rarely translate into the field. The quadruple *nigt1/hho* mutant was impaired in P_i_ uptake under P deficiency, but not when sufficient P was available (Ueda *et al.*, 2020c). Benefits in nitrate nutrition are thus compromised by negative effects on P acquisition, and crops that fail to adjust to low P_i_ are undesirable, even with improved nitrate acquisition. It is possible that complex feedback-regulated networks also help plants to explore temporal variations in nutritional availability (e.g. in field environments). As long as the underlying element is not rate limiting for growth, Arabidopsis profits from temporal reduction of external P_i_, compared with static supply (Fig. 2).

## Connecting nutrient deprivation/starvation responses with ROS

Potassium, N, or P_i_ deprivation are known to increase H_2_O_2_ in distinct areas of Arabidopsis roots; this and the misregulation of starvation-responsive nitrate and phosphate transporter genes in an NADPH oxidase mutant (*atrbohC*) provided some initial hints of ROS involvement in nutrient-specific responses (Shin *et al.*, 2005). Enforcement of the transcriptional nitrate starvation response by glutaredoxins (Jung *et al.*, 2018; Ehrary *et al.*, 2020) and inhibition by chemical ROS scavenging point to an involvement of ROS homeostasis under fluctuating nutrient conditions. This is supported by Safi *et al.* (2021), who conclude that 86% of the direct (repressed) targets of HRS1 in protoplasts were dependent on the nitrate context (then HRS1 acted mainly as a repressor of heat shock genes). In complex targeted gene categories, the redox metabolism was over-represented. H_2_O_2_ production was not compromised in a quadruple *nigt1/hho* mutant, but *HRS1/HHO1* overexpression suppressed H_2_O_2_ evolution upon nitrate deprivation. ROS signalling must not reach toxic levels within cells (Miller *et al.*, 2018) and may participate in the initiation of the response, rather than act as an unwanted by-product of long-term N starvation due to insufficient production of proteins involved in scavenging. Furthermore, *NIGT1/HHO* genes are highly specific to nitrate, rather than ammonium. Rapid responses within hours to nitrate deprivation are completely absent when ammonium is removed from the nutrient solution, and transcriptional changes are seen before nitrate levels drop in the tissue (Menz *et al.*, 2016). We therefore associate the NIGT1/HHO repressors and ROS with nitrate deprivation, rather than with N starvation responses (which occur later). Interestingly, the mRNA of *HRS1* declined quickly without nitrate, but was stabilized by NO_3_^–^, pointing to post-transcriptional control (Menz *et al.*, 2016). It may be interesting to ask if there is a link to ROS by carrying out *in silico* analysis using other datasets from previous transcriptomic studies, for example on AtNIGT1.2/AtHHO2 overexpressors (Kiba *et al.*, 2018; Wang *et al.*, 2020).

## Nutritional co-regulation is highly complex

Further crosstalk between N and P might depend on LATERAL ORGAN BOUNDARY DOMAIN transcription factors (LBD37–LBD39), another group of redundant repressors of the N deprivation response that are highly up-regulated by diverse N sources, preferentially nitrate. Their main function is to repress anthocyanin biosynthesis in nitrate-sufficient conditions. Their transcripts are repressed in –N, leading to anthocyanin coloration of shoots (and ROS scavenging) in severely –N-stressed plants (Rubin *et al.*, 2009). *LBD* transcripts may be repressed in –P (Rubin *et al.*, 2009) and less anthocyanin accumulated in the *nigt1/hho* quadruple mutant with sufficient N (Ueda *et al.*, 2020c), suggesting crosstalk with LBDs in –P (Fig. 1).

In summary, NIGT1/HHOs are central hubs in the crosstalk between nitrate and phosphate deprivation responses. How NIGT1/HHOs co-regulate these nutritional responses in crop roots and whether these can be targeted to generate more nutrient use-efficient crops is an interesting task for the future. Overexpression of various nitrate transporters increased nitrate acquisition and yield apparently independently of P, not only in laboratory studies, but even in the field (e.g. Wang *et al.*, 2018), supporting the added value of studying these genes and proteins at a fundamental level.
